# Non-Genitally Stimulated Orgasms Increase Plasma Prolactin in a Menopausal Woman

**DOI:** 10.1080/19317611.2025.2608697

**Published:** 2026-01-03

**Authors:** James G. Pfaus, Roni Erez, Nitsan Erez, Jan Novák

**Affiliations:** a Department of Psychology and Life Sciences, Faculty of Humanities, Charles University, Prague, Czech Republic; b Center for Sexual Health and Interventions, Czech National Institute of Mental Health, Klecany, Czech Republic; c The Wave Inc, Tel Aviv, Israel; d Department of Urology, General University Hospital, First Faculty of Medicine, Charles University, Prague, Czech Republic

**Keywords:** Non-genitally stimulated orgasm, hormone, prolactin, pelvic floor

## Abstract

**Background and objectives:**

Objective measures of orgasm in both women and men include increased plasma prolactin and characteristic pelvic floor contractions. Typically, these are induced by genital and other erogenous stimulation, but can occur through fantasy and willful activation of pelvic floor and abdominal muscles that likely stimulate pudendal, hypogastric, and pelvic nerves without any direct genital stimulation. It has been shown previously that non-genitally stimulated orgasms (NGSOs) from tantra result in lawful increases in prolactin in a premenopausal woman. The present study examined whether a systematic technique of pelvic floor exercises induces NGSOs that are accompanied by lawful increases in prolactin.

**Methods:**

A menopausal woman trained in a systematic technique of pelvic floor movements and able to induce orgasms fully clothed with those movements alone, agreed to induce a 2.5-min and 10-min orgasm at 48-hr intervals, along with a control condition (10-min Pilates workout). Blood samples were taken immediately before, immediately after, and 15 min after each session, and analyzed for prolactin, luteinizing hormone, follicle-stimulating hormone, and total testosterone using ELISA. Pelvic floor movements were observed during a 5-min orgasm using the Lioness 2.0 biofeedback sensor.

**Results:**

Prolactin levels increased by to 110% and 141% of baseline, respectively, after the 2.5- or 10-min NGSO. No changes were observed in the other hormones. The Pilates workout resulted in a 12% decrease in prolactin levels. The Lioness sensor revealed that orgasms came at 7- to 15-sec intervals throughout the 10-min session, and were preceded by an upswing of pelvic floor contractions.

**Conclusions:**

As with genitally-stimulated orgasms, increases in prolactin accompany NGSOs induced by specific pelvic floor movements and occur in women regardless of their reproductive status. The induction of NGSOs represents a new vista in the study of women’s orgasms and a potential therapeutic intervention for women with orgasm difficulties.

## Introduction

In the 4-stage model of human sexual response, orgasms come at the height of sexual arousal and genital stimulation, and are followed by a period of inhibition or refractoriness (Masters & Johnson, 1966; Moll, 1908/1912). The activation of opioids like β-endorphin at orgasm are responsible for the ecstatic pleasure, whereas activation of serotonin produces the longer-term satiety that characterizes refractoriness (Argiolas & Melis, 2003; Jern et al., 2023; Paredes, 2014; Pfaus, 2009, 2025). Both neurotransmitters inhibit mesolimbic and hypothalamic dopamine transmission immediately at orgasm, which in turn releases the neurohormone prolactin from tonic inhibition (Grattan, 2015). Thus, prolactin released from the anterior pituitary into the blood is an objective marker of orgasm in non-lactating individuals (Leeners et al., 2013; Pfaus & Tsarski, 2022), and its increase follows the inhibition of hypothalamic and mesolimbic dopamine; as dopamine transmission recuperates, prolactin levels fall. Orgasms are also accompanied by characteristic and intense movements of the pelvic floor and abdominal muscles. These movements can be detected by anal plethysmography in men, and by anal or vaginal plethysmography, or vaginal muscle pressure detection, in women (Bohlen et al., 1982). The spinal and brainstem control of this motor reflex characterizes the feelings of release that characterize “climax,” whereas the conscious awareness of pleasurable sensations that characterize orgasm involves the activation of opioids, serotonin, and other neurotransmitters like oxytocin in the brain (McKenna, 2022; Pfaus, 2025).

Orgasms can also be induced without genital stimulation (Herbenick et al., 2021; Kanzer, 1954; Money, 1960; Pfaus, 2025; Pfaus & Tsarski, 2022; Wells, 1986; Whipple et al., 1992). Accordingly, non-genitally stimulated orgasms (NGSOs) occur during sleep, after exercise, or as a result of fantasy or tactile stimulation of erogenous skin (e.g. nipples, ears, toes). This suggests a “top-down” cognitive control of orgasm that can be activated on its own under certain conditions in sensitized individuals. The ability to generate NGSOs can also come as a result of tantric training in which individuals learn to control their abdominal and pelvic floor muscles during deep breathing. Some of these individuals experience spontaneous NGSOs while doing so.

A previous study examined NGSOs induced in a premenopausal woman who had developed the ability to place herself into a continuous orgasmic state through tantric training (Pfaus & Tsarski, 2022). She had her blood analyzed 15 min before, immediately after, and 15 min after a 5-min or 10-min orgasm, relative to a non-orgasmic control condition (10 min of book reading). Tests were run at weekly intervals in a hospital examination room while the participant was fully clothed. Blood draws were taken by a registered nurse and plasma was analyzed by ELISA for prolactin, luteinizing hormone (LH), follicle-stimulating hormone (FSH), and free testosterone. Prolactin levels from Pre to Post increased to 125% or 148%, respectively, after the 5- or 10-min NGSO, and were still elevated from the Pre-baseline 15 min after. No changes were recorded in FSH or free testosterone. Interestingly, LH levels were higher to begin with on the day of the 5-min NGSO (in a range suggesting a preovulatory surge), and the NGSO increased her LH levels to 145%, which remained elevated 15 min after. This elevation did not occur a week later when the woman was in her luteal phase and the 10-min NGSO was evaluated. No effects on prolactin or other hormones were detected before, during, or after the non-orgasmic control. Subjectively, her NGSOs were rated as pleasurable as genitally stimulated orgasms (GSOs) induced by direct external or internal clitoral stimulation. Being a single case of a premenopausal woman, it was not known whether this ability required circulating ovarian steroids. In addition, although it was obvious during the orgasmic inductions that both the abdominal and pelvic floor muscles were contracting in circular spasms throughout the 5- or 10-min test period, no recording of those muscle movements was made in that study.

## Case study

The present case study involved a 55-year-old menopausal woman who had a complete hysterectomy without oophorectomy 12 years prior to the experiment and was not on hormone replacement. She learned to induce NGSOs by engaging in a set of pelvic floor movements called “The Wave Technique.” This technique involves a programmatic set of intense Kegel-like exercises that sensitize pelvic floor contractions and flexions (pulling, pushing, and relaxing). With the use of a small jade egg in the vagina, women learn to move the egg upward from the introitus of the vagina to the clitorourethrovaginal complex (CUV complex, formerly called the “G-spot;” Jannini et al., 2014) and back down again in a circular motion. Eventually, women can move the same muscles without the jade egg, and this reliably sensitizes the ability to induce NGSOs from pelvic floor contractions alone.

We modified the methodology used in the study by Pfaus and Tsarski (2022) to examine the prolactin response 15 min before (Pre), immediately after (Post), and 15 min after (After) a 2.5-min NGSO, a 10-min NGSO, or a 10-min Pilates workout as the control (since it involved non-orgasmic movements of the abdominal and pelvic floor muscles). In addition, we examined her pelvic floor movements during a 5-min NGSO using the Lioness 2.0 Bluetooth device. This device detects the force of pelvic floor contractions in two sensors at either side of the instrument that collect continuous pressure (in grams force) at a sampling rate of 12 Hz. It connects by Bluetooth to a secure internet server from which users can download their pelvic floor output onto an app. As in the previous study, tests were conducted while the participant was fully clothed. However, in this study, the participant was able to lie on a comfortable bed in a private room while inducing her NGSOs. At the beginning of each experimental session, the participant signed informed consent, then sat in a comfortable chair and had a 23-gauge butterfly IV catheter inserted into the medial cubital vein of her nondominant arm, whereupon the first blood draw (Pre) was taken 15 min before the participant went into the private room. She was instructed to relax and, when she felt ready to begin, to start a timer. When the timer rang at the end of 2.5 or 10 min, the participant was instructed to spend a few seconds calming down, then to exit the room, sit in the armchair, and have the second (Post) blood draw. She then sat in the armchair for another 15 min until the final (After) blood draw was taken. For the 10-min Pilates workout, the participant brought a mat and did a routine workout of her own design. All tests were overseen by a board-certified urologist and sexual medicine specialist, and the study had received ethics approval from the Human Ethics Research Committee of the Czech National Institute of Mental Health (NUDZ c.j. 58/24).

Tests were run every 48 hr and blood draws (5 ml) were taken by a registered nurse. The order was 2.5-min NGSO, 10-min NGSO, Pilates workout, and 5-min NGSO with pelvic floor detection (no blood was taken during that test). All tests began at 10:00 hr. The 3 blood samples were picked up at the end of each morning’s test by a commercial laboratory (SynLab, Prague 6, www.synlab.cz) and analyzed immediately for prolactin (mUI/l), LH (IU/l), FSH (IU/l), free testosterone (nmol/l), SHBG (nmol/l), cortisol (nmol/l), and serum albumin (g/l) using an enzyme linked immunosorbent assay (ELISA). The lab then sent the data to the participant (second author) who coded it so that the first author would be blind to the experiential conditions. After values were plotted graphically, the code was broken to reveal each condition.

**Figure 1.**
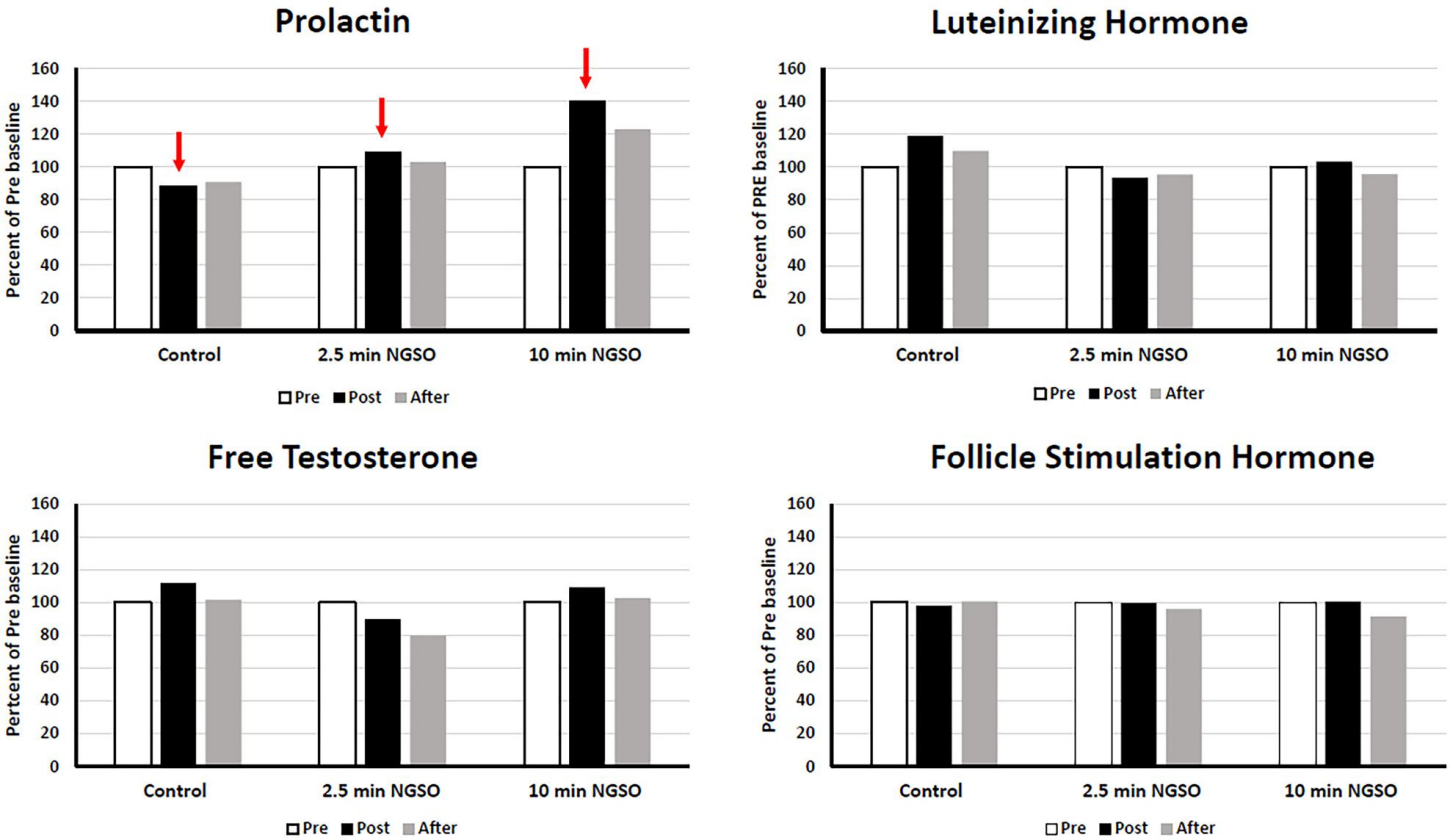
Serum levels of prolactin, luteinizing hormone, total testosterone, and follicle-stimulating hormone as a percent change from the pre-baseline during the three conditions (control, 2.5-min NGSO, 10-min NGSO).

**Figure 2.**
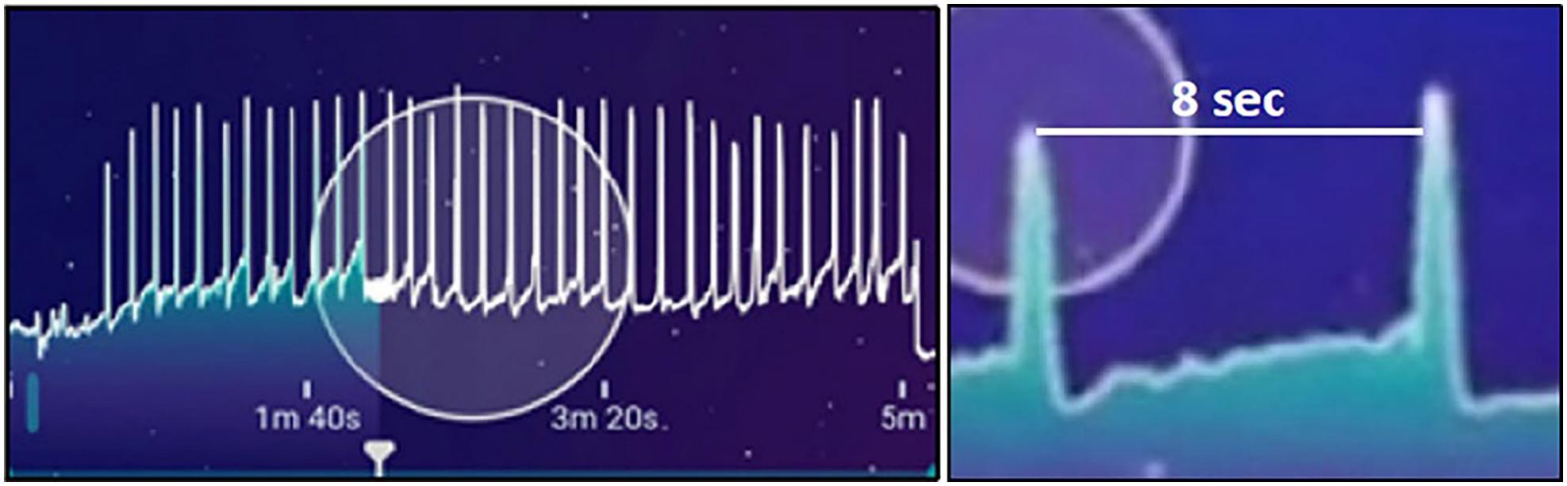
Left: “Wave”-like orgasms (spikes) came at 6–12 sec intervals during the 5-min NGSO. Right: Inter-orgasm intervals showed quick push and pull contractions that increased pelvic floor tension leading to orgasms. Output is pelvic floor muscle pressure (in grams of force) detected by dual sensors in the Lioness 2.0.

Prolactin levels from Pre to Post increased to 111% or 140% of baseline during the 2.5- or 10-min NGSO, respectively, and were still elevated from the Pre-baseline 15 min after in the case of the 10-min NGSO (Figure 1). Testosterone was elevated to 114% of baseline after the 10-min NGSO, but decreased after the 2.5-min NGSO. None of the other hormones changed as a result of orgasm. During the Pilates exercise control, prolactin dropped to 88% of baseline. However, both LH and total testosterone were elevated after the Pilates workout (to 120% and 116% of baseline, respectively). For the pelvic floor muscle activation experiment, the clitoral stimulator arm of the Lioness 2.0 had to be tied back with duct tape so that it would not generate any tactile stimulation of the clitoris. The participant was instructed to lie back and insert the Lioness detector comfortably into the vagina and, when she was ready, to start a timer and induce a 5-min NGSO. The participant did this alone in the same testing room with the door locked. The Lioness detected “wave”-like orgasms (spikes) that came at 6–12 sec intervals during the 5-min NGSO (Figure 2). A total of 34 spikes occurred during this period. Inter-orgasm intervals showed quick push and pull contractions that increased pelvic floor tension progressively leading to each orgasm. The participant was heard vocalizing during each spike that ended the upswing. The participant stated that after that, she could feel the Lioness in her vagina, but that it had not altered her ability to induce the NGSOs from her pelvic floor movements.

## Discussion

The results of this case study indicate that NGSOs induced by programmatic pelvic floor contractions in a menopausal woman increase prolactin in a specific and lawful manner without altering the levels of other hormones. This indicates that circulating ovarian steroids are not necessary for the induction of an NGSO. The present study also utilized a 48-hr separation between tests, rather than a week separation, with no decrement in baseline prolactin levels from test to test. The pelvic floor contractions that induced orgasms came at relatively regular intervals and had a characteristic upswing prior to each spike/orgasm. These data suggest strongly that NGSOs are not “faked,” but rather reflect a top–down induction of a real orgasmic state that includes objective increases in prolactin and characteristic pelvic floor contractions. The finding of increased LH and free testosterone after the Pilates exercise is consistent with previous reports that acute exercise elevates both ovarian and adrenal androgens in women (Enea et al., 2011), albeit to a small extent in the present study. Thus, the slight increase in testosterone during the NGSO induction may have also been due to general physical exertion.

## Conclusion

As with GSOs from clitoral, CUV, and/or cervical stimulation, lawful increases in prolactin accompany NGSOs in women. NGSOs can be induced in women by entrained pelvic floor muscle contractions that likely stimulate pudendal, hypogastric, and/or pelvic nerve fibers directly. NGSOs can be produced by fantasy, stimulation of erogenous zones, hypnosis, and by rhythmic “circular” contracting and flexing of the pelvic floor musculature. Being able to study orgasms in fully clothed participants represents a new vista in the study of women’s orgasms. Finally, the programmatic method of induction used in the present study has potential as a therapeutic intervention for women with orgasm difficulties.
